# Climate change mitigation strategies in the forest sector: biophysical impacts and economic implications in British Columbia, Canada

**DOI:** 10.1007/s11027-016-9735-7

**Published:** 2017-02-17

**Authors:** Zhen Xu, Carolyn E. Smyth, Tony C. Lemprière, Greg J. Rampley, Werner A. Kurz

**Affiliations:** 1 Faculty of Forestry, Department of Forest Resources Management, 2045–2424 Main Mall, University of British Columbia, Vancouver, BC V6T 1Z4 Canada; 2 Natural Resources Canada, Canadian Forest Service, 506 Burnside Road West, Victoria, BC V8Z 1M5 Canada; 3 Natural Resources Canada, Canadian Forest Service, 606–55 St. Clair Ave. East, Toronto, ON M4T 1L8 Canada; 4 Natural Resources Canada, Canadian Forest Service, 580 Booth Street, Ottawa, ON K1A 0E4 Canada

**Keywords:** Climate change mitigation strategy, Forest sector, Mitigation cost, Socio-economic impact, CBM-CFS3

## Abstract

Managing forests to increase carbon sequestration or reduce carbon emissions and using wood products and bioenergy to store carbon and substitute for other emission-intensive products and fossil fuel energy have been considered effective ways to tackle climate change in many countries and regions. The objective of this study is to examine the climate change mitigation potential of the forest sector by developing and assessing potential mitigation strategies and portfolios with various goals in British Columbia (BC), Canada. From a systems perspective, mitigation potentials of five individual strategies and their combinations were examined with regionally differentiated implementations of changes. We also calculated cost curves for the strategies and explored socio-economic impacts using an input-output model. Our results showed a wide range of mitigation potentials and that both the magnitude and the timing of mitigation varied across strategies. The greatest mitigation potential was achieved by improving the harvest utilization, shifting the commodity mix to longer-lived wood products, and using harvest residues for bioenergy. The highest cumulative mitigation of 421 MtCO_2_e for BC was estimated when employing the strategy portfolio that maximized domestic mitigation during 2017–2050, and this would contribute 35% of BC’s greenhouse gas emission reduction target by 2050 at less than $100/tCO_2_e and provide additional socio-economic benefits. This case study demonstrated the application of an integrated systems approach that tracks carbon stock changes and emissions in forest ecosystems, harvested wood products (HWPs), and the avoidance of emissions through the use of HWPs and is therefore applicable to other countries and regions.

## Introduction

Forests are essential for global climate change mitigation, because they can contribute carbon sinks. Forests not affected by land-use change remove about 8.8 GtCO_2_e/year from the atmosphere (Pan et al. 2011). The Intergovernmental Panel on Climate Change (IPCC) reported that the forest sector has a mitigation potential of 0.2–13.8 GtCO_2_e/year in 2030 with a cost up to US$100/tCO_2_e (Smith et al. 2014). At the global scale, among various mitigation strategies, the use of wood products and wood energy represents high mitigation potential, as well as afforestation and reforestation options (IPCC 2014). Many studies have examined the potential of the forest sector for climate change mitigation at both global and regional scales (Kurz and Apps 1995; Bourque et al. 2007; Nabuurs et al. 2007; Lippke et al. 2011). FAO (2016) highlighted that, globally, forest activities can provide economic mitigation potential ranging from 1.9 to 5.5 GtCO_2_e/year in 2040 at costs less than US$20/tCO_2_e. Two background studies for FAO (2016) indicated that wood products, especially panels, also play an important role in climate change mitigation because of substitution effects. Seidl et al. (2007) examined mitigation impacts of alternative silviculture strategies in a forest management unit in Austria. Lundmark et al. (2014) compared two forest management and wood use strategies to a “do-nothing” scenario from a systems perspective in Sweden. Werner et al. (2010) investigated various forest and wood strategies using an integral model-based approach in Switzerland. In Canada, Smyth et al. (2014) conducted the first comprehensive study examining the mitigation potential of Canada’s managed forest and harvested wood products (HWPs). However, there are few studies that provide quantitative analyses of the mitigation potential of the forest sector in response to a series of possible mitigation strategies at national or provincial scale with considerable spatial detail. In fact, a thorough assessment of the impacts of mitigation strategies is complex due to the interaction between the forest sector and energy and other industrial product sectors, and a systems perspective is required to consider the carbon flow within and among forest ecosystems, wood products, and displacement effects when substituting wood-based products and energy for emission-intensive products (e.g., concrete, steel, plastic, etc.) and fossil fuel energy (Nabuurs et al. 2007; Lemprière et al. 2013; Smyth et al. 2014; Kurz et al. 2016).

The economically feasible mitigation benefits are expected to be lower than the biophysical mitigation potential, because the implementation of mitigation strategies has technical and economic constraints (the society’s resources that can be devoted to climate change mitigation are limited). It is therefore necessary to analyze the cost of mitigation strategies (Boyland 2006; Nabuurs et al. 2007; Strengers et al. 2008; van Minnen et al. 2008; Lemprière et al. 2013). The economics of forest carbon mitigation strategies including costs have been examined in Canada for activities such as afforestation (McKenney et al. 2004; Yemshanov et al. 2005; Yemshanov et al. 2015), harvest reduction (Man et al. 2015), bioenergy (Stennes and McBeath 2006; Ralevic et al. 2010; Zhang et al. 2010), intensive forest management (Insley et al. 2002), and for multiple strategies (van Kooten et al. 1992; Krcmar and van Kooten 2005; Kennedy et al. 2007). Based on the results of Smyth et al. (2014), Lemprière et al. (2017) conducted the first in-depth national examination of the economics of mitigation strategies from a systems perspective. Although at a coarse spatial resolution, they found that some strategies are likely to provide substantial cost-effective mitigation over the medium and long term to meet Canada’s emission reduction targets, provided that mitigation actions are initiated soon.

In this study, we further refined the methodology of Smyth et al. (2014) and Lemprière et al. (2017) with a much finer spatial resolution and applied it to the forest sector in the Canadian province of British Columbia to demonstrate the biophysical and economic mitigation potentials in response to a range of regionally differentiated strategies and portfolios. Socio-economic impacts of mitigation strategies were incorporated in the economic analysis to capture implications on the British Columbia’s (BC’s) economy and social welfare. The primary objective of this study was to quantify and compare the biophysical, economic, and socio-economic impacts of various mitigation strategies for BC to a business-as-usual scenario. BC’s forests and forest sector are among the most significant in Canada, and its forests are managed to achieve a balance across multiple goals including habitat, sustainable harvesting, employment, and others (Hoberg et al. 2016): contributing to climate change mitigation could become another goal to include in the balance. This study does not consider these other goals.

## Methods

### Analytical framework

BC’s forests cover about 55 million hectares, of which 95% are owned by the provincial government. BC is the largest exporter of softwood lumber in the world and the largest bioenergy producer in North America. It is estimated that about 6–7 billion tonnes of carbon are stored in the aboveground biomass (95% of which are certified by third-party certification) (BCMoFLNRO 2013), with an average net carbon removal from the atmosphere of 62.8 MtCO_2_e/year during the last 25 years (Government of British Columbia 2016), which is equivalent to the total annual CO_2_e emissions from all other sectors in BC. Harvesting of BC’s forests transfers roughly the same amount of carbon (66.2 MtCO_2_e/year) to wood products. These characteristics suggest that the mitigation potential in BC’s forest sector could be substantial if appropriate strategies are implemented.

**Fig. 1:**
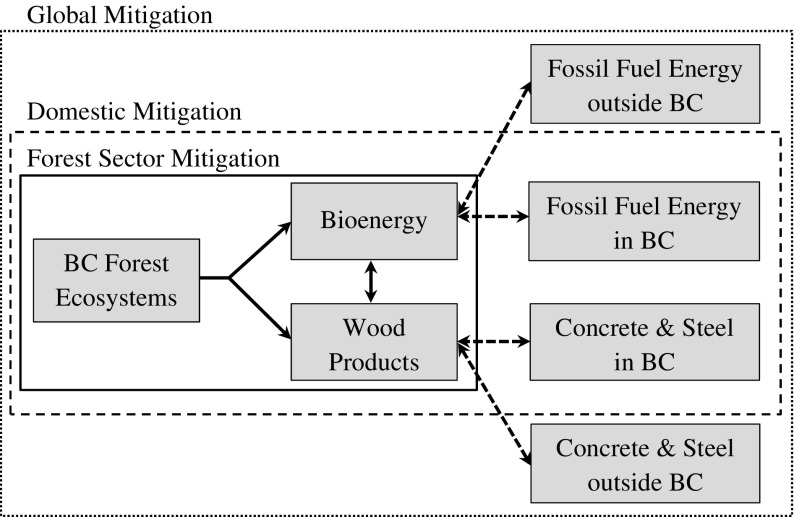
A systems perspective that includes multiple sectors. The *solid arrows* refer to carbon flows within the forest sector, and the *dashed arrows* represent substitution effects between biofuel and fossil fuel and between long-lived products and concrete/steel (adapted from IPCC 2007, Fig. 9.3)

In line with the IPCC’s definition of mitigation (IPCC 2014), our analysis considered mitigation potential as reduced greenhouse gas (GHG) emission or enhanced carbon sequestration that would result from implementation of a mitigation option, relative to a baseline. Such an approach canceled out all factors that were assumed to be unchanged between the baseline and a mitigation scenario, including variables with uncertainties like GHG emissions from wildfires. A systems perspective was employed to include potential mitigation resulting from changes in forest management, use of longer-lived products (LLPs) and bioenergy, as well as avoided emissions in other sectors due to displacement effects (Fig. 1). We defined forest sector mitigation based on carbon stock changes in BC’s forest ecosystems and in harvested wood products manufactured from wood that was harvested in BC regardless of where in the world these products reside—the IPCC production approach for estimation of HWP C balances (IPCC 2013). Domestic mitigation is the sum of forest sector mitigation plus displacement effects in BC resulting from the use of BC harvested wood products. Global mitigation is the sum of domestic mitigation plus the displacement effects that occur outside BC as a consequence of the use of HWP manufactured from wood harvested in BC. All displacement factors assumed that concrete and steel would be used as an alternative to BC wood, rather than wood from elsewhere. At all scales, we did not consider possible leakage effects due to, for example, displacement from imported wood products, or imperfect substitution due to market interactions—we focused on the mitigation that BC’s forest sector may contribute rather than the net mitigation benefit for the global atmosphere. The exclusion of leakage effects in this analysis is justified by the scale of our analysis. Leakages from shifting harvest and land-use change need to be considered at the scale of individual offset projects but are expected to be minimal at the provincial scale, because changes resulting from mitigation strategies were small compared to the size of BC’s forest sector.

**Fig. 2:**
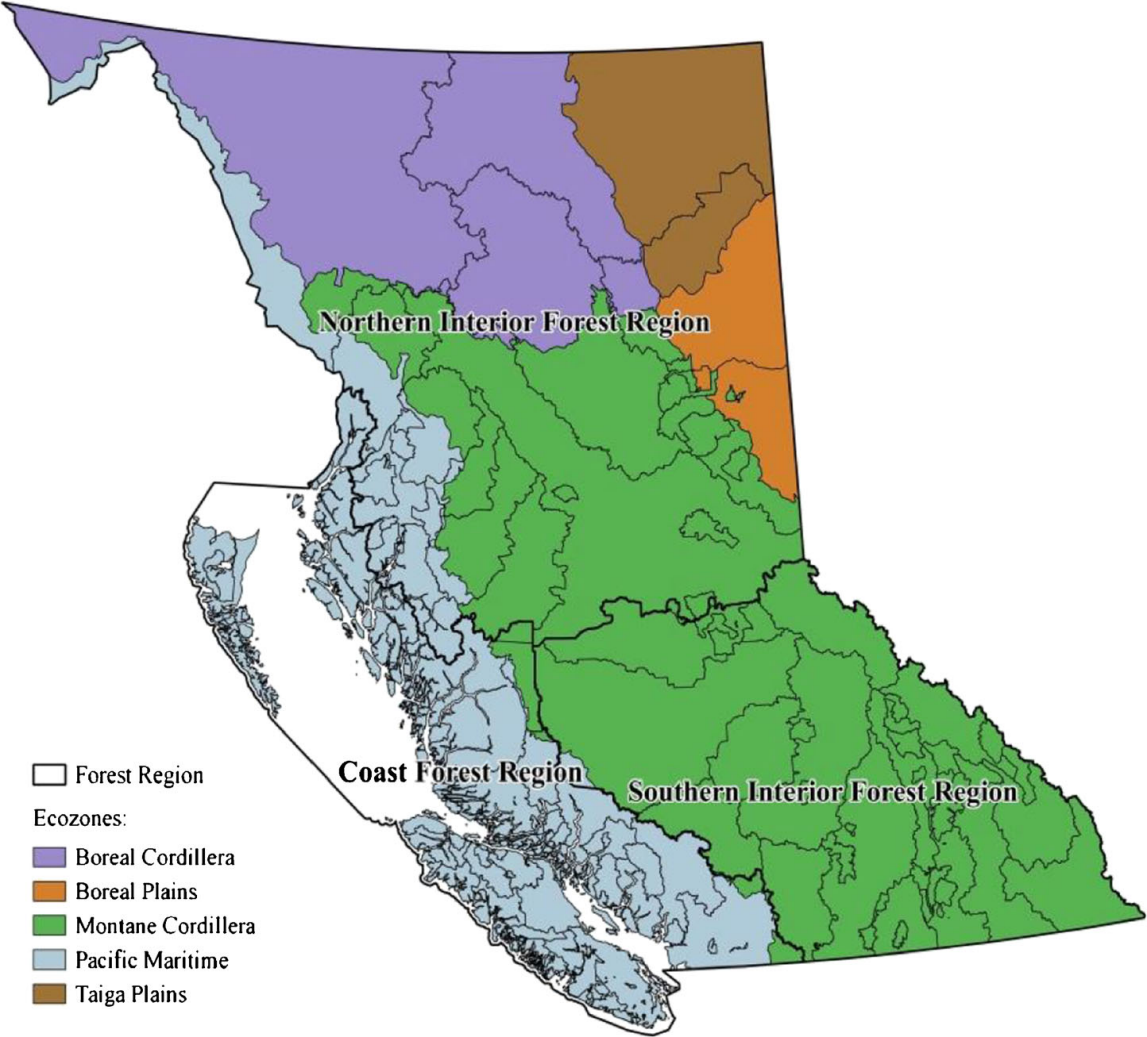
Forest management units categorized by ecozones (*colors*) and forest regions (*thick lines*)

Our analysis was conducted at a spatial resolution with 74 spatial units based on the forest management units (FMUs) identified in Canada’s 2014 National GHG Inventory Report (Environment Canada 2016). These FMUs in BC were defined by the boundaries of Timber Supply Areas (TSA) and Tree Farm Licences (TFL) and categorized in five ecozones and three forest regions (Fig. 2). The mitigation potential in each FMU was examined for the period from 2017 to 2050, within which three periods were of particular interest in terms of BC’s and Canada’s GHG emission reduction targets: 2017–2020 (short term), 2017–2030 (medium term), and 2017–2050 (long term).

In this study, eleven different strategies were assessed relative to the baseline, including five individual strategies and six combinations of the individual strategies (Table 1). The mitigation strategies were developed in consultation with experts and professionals from the BC Ministry of Forests, Lands, and Natural Resource Operations (BCMoFLNRO), the BC Ministry of Environment, and other organizations. Although some of the strategies are nominally similar to those in the national study of Smyth et al. (2014), all strategies in this study were adjusted based on the biophysical and economic characteristics of BC’s forest sector, as well as the province’s mitigation needs (see “Appendix” for further details of the five individual strategies). Some strategies were implemented with a ramp-up period of 2017–2020 as it was expected that time would be needed to scale-up efforts.

**Table 1:** Individual mitigation strategies and their combinations

Individual strategy	Implementation (relative to baseline assumptions)
Higher utilization	• Increase merchantable utilization - 2017–2020: from 85 to 86.5% - 2021–2050: from 86.5 to 90% •Increase salvage harvesting - 2017–2020: from 6 to 7% - 2021–2050: from 7 to 10%
Harvest less	•Reduce harvest volume by 2% from 2017 to 2050
Harvest residue for bioenergy	•Collect harvest residues for local bioenergy production - 2017–2020: reduce the proportion of slashburning from 50% of total harvest area to 40% and transfer 10% harvest residue carbon to bioenergy - 2021–2050: reduce the proportion of slashburning from 40% of total harvest area to 25% and transfer 25% harvest residue carbon to bioenergy
Restricted harvest	•Restrict harvest to stands less than 250 years old from 2017 to 2050
More longer-lived products	•Shift the commodity mix from pulp and paper to panels - 2017–2020: reduce harvest used for pulp and paper by 1.6% and increase harvest used for panels by 1.6% - 2021–2050: reduce harvest used for pulp and paper by 4% and increase harvest used for panels by 4%
Strategy combination	Additivity^a^
Higher utilization + harvest residue for bioenergy	Not additive
Higher utilization + more LLP	Additive
Harvest less + more LLP	Not additive
Harvest residue for bioenergy + more LLP	Additive
Restricted harvest + more LLP	Not additive
Higher utilization + harvest Residue for bioenergy + more LLP	Not additive

^a^Additivity indicates whether the individual strategies in a combination interact with each other. If additive, effects of individual strategies can be added together to determine the effect of the strategy combination. If not, the combination was modeled as a combination strategy

The baseline was defined as the forest management activities and use of HWP that would occur in the absence of mitigation activities. Modeling assumptions for the historical period (1990–2012) in the baseline were based on Canada’s GHG National Inventory Report (Environment Canada 2016). We included harvest and wildfire projections for each FMU for the future time period (2013–2050) based on a forecast of future harvest levels and historical average annual area burned during 1990–2012, respectively.

Mitigation effects for each strategy are expected to vary across FMUs depending on various factors, such as size of the spatial unit, forest characteristics, harvest levels, potential for substitution of fossil fuels, and logging and transportation costs. Implementation of the strategies was modeled for each FMU independently, i.e., there were no interactions between FMUs. We recognized that there is no “best-for-all” strategy for the province; rather, a portfolio of strategies with FMU-specific strategy selections would generate greater mitigation and/or require lower cost. Therefore, we first estimated attributes of interest (e.g., global, domestic or forest sector mitigation, or mitigation cost) for all strategies in each FMU and then constructed a specific portfolio by selecting the strategy to best meet the portfolio goal in each FMU over a specific time period. Among others, four portfolios for the long term (2017–2050) were of particular interest: (1) portfolio that maximizes global mitigation (PORT1), (2) portfolio that maximizes domestic mitigation (PORT2), (3) portfolio that maximizes forest sector mitigation (PORT3), and (4) portfolio that minimizes domestic mitigation cost (PORT4) (see Fig. 5 for all portfolios).

### Mitigation impact of strategies

Forest ecosystem carbon dynamics in this study were estimated using the Carbon Budget Model of the Canadian Forest Sector (CBM-CFS3) (Kurz et al. 2009) with historical datasets for BC from the National Forest Carbon Monitoring, Accounting and Reporting System (Kurz and Apps 2006; Stinson et al. 2011). Carbon transferred from forest ecosystems to HWP and bioenergy were tracked through manufacturing, use/export, and end-of-life use by the Carbon Budget Modeling Framework for Harvested Wood Products (Smyth et al. 2014). More details about the carbon models are described in the “Appendix”.

Following Smyth et al. (2014), we defined mitigation as the difference in GHG emissions between the baseline and a scenario with mitigation actions:

$$ M={E}_{\mathrm{base}}-{E}_{\mathrm{strategy}} $$

where *M* is the mitigation and *E*_base_ and *E*_strategy_ are the net emissions in the baseline scenario and the mitigation scenario, respectively. In both scenarios, net emissions included three components—the forest ecosystem emissions, the HWP emissions including bioenergy, and emissions as a result of displacement. Displacement impacts for products included the emissions associated with the extraction and transportation of raw materials and the manufacturing of products, based on aggregated studies in the literature (Smyth et al. 2016).

To obtain the total cumulative global mitigation impact of a strategy, we aggregated FMU results for the strategy to the provincial level. Emissions associated with exported HWPs were taken into account in order to be consistent with the IPCC Production Approach in accordance with internationally agreed guidance (IPCC 2013). Displacement effects that occurred abroad were included in the global mitigation impact of a strategy but not in the domestic mitigation impact which was used for economic analyses, as mitigation resulting from displacement occurring abroad is not included in Canada’s national or provincial GHG inventories (Environment Canada 2016). We also assumed that bioenergy from harvest residues was produced and consumed only within BC.

Two displacement effects were considered in all individual strategies and associated combinations involving a change in HWP or bioenergy production: substitution between solid wood products (panels) and emission-intensive materials (concrete and steel) in housing construction and substitution between bioenergy from harvest residues and fossil fuel energy in providing power and heat. Emissions related to displacement were calculated by employing displacement factors for sawnwood, panels, and bioenergy. Displacement factors are commonly used to indicate how many tonnes of carbon emissions from alternatives can be avoided per tonne of carbon in wood-based products used (Sathre and O’Connor 2010). In this study, we applied national average displacement factors of 2.1 tC/tC for sawnwood and 2.2 tC/tC for panels derived from Smyth et al. (2016) for the entire period by considering three end-use products: single-family home, multi-family home, and multi-use building. For avoided emissions for bioenergy in BC, we estimated displacement factors using a linear programming (LP) model that maximized avoided emissions in each FMU by selecting different bioenergy facilities to substitute for the most emission-intensive fuel sources that would have been used for baseline electricity and heat generation. Smyth et al. (2016) provided a detailed description of the LP model, and details of the bioenergy facilities are summarized in Table 2.

**Table 2:** Bioenergy facility types and characteristics adapted from Smyth et al. (2016)

Type	Scale	Description	Biomass demand (kodt/year)	Electrical conversion rate (MWh/odt)	Thermal conversion rate (GJ/odt)	Assumed electrical efficiency (%)	Assumed thermal efficiency (%)	Implied overall efficiency (%)	Production cost^f^ ($/MWh)
Heat	Small	0.4 MWth boiler for district heating^a^	0.783	–	15.0	–	75	75	13.28
	Medium	2.3 MWth boiler for district heating^a^	3.97	–	17.0	–	85	85	9.99
	Large	6.62 MWth process heat via syngas^b^	11.58	–	16.8	–	84	84	7.12
Power	Small	0.2 MWe gas turbine^c^	1.60	1.02	–	18	–	18	173.09
	Medium	5 MWe steam cycle^b^	34.97	1.17	–	21	–	21	34.70
	Large	10 MWe steam cycle^b^	63.86	1.28	–	23	–	23	30.15
CHP	Small	0.2 Mwe, 0.98 MWth organic rankine cycle^d^	2.09	0.78	14.0	14	70	84	123.17
	Medium	1.8 MWe,4.5MWth steam turbine^e^	10.58	1.39	10.8	25	54	79	63.36
	Large	8 MWe CHP steam turbine^b^	46.87	1.39	5.88	25	29	54	56.43

^a^RETScreen International (2015)

^b^Biopathways (FPAC and FPInnovations 2011)

^c^Arena et al. (2010)

^d^Wood and Rowley (2011)

^e^Pröll et al. (2011)

^f^Production costs (2008 dollars) include fiber costs described in Table 7

### Mitigation costs of strategies

The domestic mitigation costs (Canadian dollars) were estimated using the Model for Economic Analysis of Forest Carbon Management (MEA-FCM) which was originally designed and employed by Lemprière et al. (2017). We only considered domestic mitigation rather than global mitigation, because mitigation costs associated with BC’s forest sector and related local industries were of interest in this study. We defined mitigation cost (*TC*) as the total cost to implement a mitigation strategy which equals the change between the baseline and a mitigation scenario in the present values of the total net revenues (*NR*) of both the forest sector and the other industries/sectors affected by displacement:

$$ TC=\varDelta {NR}_{\mathrm{forest}}+\varDelta {NR}_{\mathrm{dis}} $$

where Δ*NR*_forest_ is the total net revenue change in the forest sector, and Δ*NR*_dis_ is the total net revenue change in other industries/sectors. The total net revenues of the forest sector (*NR*_forest_) in either the baseline or a mitigation scenario can be further broken down as follows:

$$ {NR}_{\mathrm{forest}}={R}_{\mathrm{fm}}-{C}_{\mathrm{fm}}+{R}_{\mathrm{hwp}}-{C}_{\mathrm{hwp}} $$

where *R*_fm_ refers to total revenue in forest management via harvesting, and *R*_hwp_ refers to total revenue from wood product manufacturing and bioenergy production; and *C*_fm_ and *C*_hwp_ are the associated total costs. The total revenue change in the forest sector was calculated by taking the differences in all components between the baseline and a mitigation scenario.

For the total net revenue change in other industries/sectors, we considered concrete and steel industries and part of the energy sector that generates electricity and heat using fossil fuels:

$$ \varDelta {NR}_{\mathrm{dis}}=\left({p}_{\mathrm{c}}-{c}_{\mathrm{c}}\right)\times \varDelta {Q}_{\mathrm{panel}}\times {u}_{\mathrm{c}}+\left({p}_{\mathrm{s}}-{c}_{\mathrm{s}}\right)\times \varDelta {Q}_{\mathrm{panel}}\times {u}_{\mathrm{s}}+\left({p}_{\mathrm{e}}-{c}_{\mathrm{e}}\right)\times \varDelta {Q}_{\mathrm{residue}}\times {u}_{\mathrm{e}} $$

where *p*_c_, *p*_s_, and *p*_e_ and *c*_c_, *c*_s_, and *c*_e_ refer to the per unit prices and costs of concrete and steel products, as well as fossil fuel energy, respectively; *u*_c_ and *u*_s_ are parameters that indicate how many tonnes of concrete and steel can be substituted per cubic meter of panels, respectively, and *u*_e_ is the parameter that indicates the amount of bioenergy (MWh) that can be produced per cubic meter of captured harvest residues. Bioenergy displaced power and heat for residential and industrial uses, and for simplicity, we measured both energy types using MWh. Δ*Q*_panel_ and Δ*Q*_residue_ are the volume changes in panel production and harvest residues between the baseline and the strategies. Note that *u*_c_ and *u*_s_ are constants while *u*_e_ varied across FMUs as each had a different fuel mix that was displaced by harvest residues. To be consistent with the calculation of displacement factors, we assumed that all concrete and steel products are domestically produced in BC—we included net revenue changes in those two industries in the mitigation cost, though we realized that most steel used in BC is imported and thus the profit changes in the steel industry actually occur outside of BC.

The cost per tonne ($/tCO_2_e) of domestic mitigation in each FMU for each strategy was then calculated by dividing mitigation cost by domestic mitigation impact over the time period:

$$ {MC}_{ni}=\frac{PTC_{ni}}{PDE{}_{ni}} $$

where *MC*_*ni*_ is the cost per tonne in FMU *i* for strategy *n*, *PTC*_*ni*_ is the present value (2016 as the base year) of the total mitigation cost during 2017–2050 with a 3% discount rate, and *PDE*_*ni*_ is the present value of the change in total domestic emissions between the baseline and the strategy scenario with a 1% discount rate. The 3% real social discount rate was selected based on the Canadian Cost-Benefit Analysis Guide (TBS 2007). The 1% discount rate for carbon emissions was derived from the social discount rate adjusted for the marginal social cost of damages resulting from emissions—we assumed that physical carbon emissions are a proxy for the social cost of the damages, and the marginal damage of emissions will grow at an annual rate of 2% (Greenstone et al. 2013). Therefore, a 3% social discount rate for monetary value and a 2% rate for marginal damage of emissions imply a 1% discount rate for carbon emissions. By discounting both mitigation cost and quantity, cost per tonne of mitigation was considered a measure of cost effectiveness of a mitigation strategy over the entire period of 2017–2050. We kept the 2% difference between the discount rates for monetary values and the carbon emissions in the sensitivity analysis to keep the marginal damage of emission unchanged over time.

For the prices and costs for harvesting and products, we used annual averages to reflect long-term trends and assumed that they did not change over time. Detailed assumptions were developed in consultation with BCMoFLNRO and FPInnovations and summarized in Tables 3, 4, 5, and 6. Softwood/hardwood log costs in the baseline were estimates derived from log cost surveys. The log cost includes tree-to-truck cost, hauling cost, cost of stumpage, and costs for forest planning and administration, road development and management, and silviculture. Prices and costs for salvage logging and other industrial roundwood were assumed to be the same as those for regular harvest and sawnwood, respectively. The higher utilization strategy was assumed to slightly decrease the log cost per cubic meter, because the harvest volume was kept unchanged but the harvested area was smaller. The harvest less strategy reduced the harvest volume and was assumed to increase the logging cost since cut blocks were assumed to be more dispersed in order to match the same timber characteristics as in the baseline. A similar assumption was made for the restricted harvest strategy as more young stands would be harvested to meet the target harvest level. No price/cost changes in harvesting were assumed in the harvest residue for bioenergy (hereafter bioenergy) strategy.

**Table 3:** Harvest cost and price assumptions for individual strategies ($/m^3^ in 2014 dollars)

Scenario	Forest region	Softwood log price^a^	Hardwood log price^a^	Salvage log price	Softwood log cost^b^	Hardwood log cost^b^	Salvage log cost
Base case	Northern interior	53	43	43	48	38	38
	Southern interior	57	46	46	52	41	41
	Coast	86	76	76	81	71	71
Higher utilization	Northern interior	No change			$0.2/m^3^ decrease^c^		No change
	Southern interior						
	Coast				$0.25/m^3^ decrease^c^		
Harvest less	Northern interior	No change			$0.37/m^3^ increase^c^		No change
	Southern interior						
	Coast						
Harvest residue for bioenergy	Northern interior	No change					
	Southern interior						
	Coast						
Restricted harvest	Northern interior	No change			$0.45/m^3^ increase^c^		
	Southern interior				$0.48/m^3^ increase^c^		
	Coast				$1.82/m^3^ increase^c^		

^a^Log market reports (2008–2015 averages), BCMoFLNRO. http://www2.gov.bc.ca/gov/content/industry/forestry/competitive-forest-industry/timber-pricing

^b^Based on personal communication with BCMoFLNRO (Ryan Midgley, January 8, 2016)

^c^Based on personal communications with FPInnovations (Denis Cormier and Jean Favreau, July 25, 2011)

**Table 4:** Price assumptions for individual strategies for harvested wood products and bioenergy (2014 dollars)

Scenario	Forest region	Sawnwood price ($/m^3^)^a^	Panel price ($/m^3^)^b^	Other industrial roundwood price ($/m^3^)	Pulp price ($/odt)^c^	Bioenergy price ($/MWh)
Base case	Northern interior	130	238	130	854	–
	Southern interior	130	238	130	854	
	Coast	130	238	130	854	
Harvest residue for bioenergy	Northern interior	No change				Varies spatially
	Southern interior					
	Coast					
More longer-lived products	Northern interior	No change				–
	Southern interior					
	Coast					

^a^Lumber average sales price for BC Interior during 2005–2014, Forest Economic Advisors LLC

^b^OSB (7/16 in.) average sales price for US Western Canada during 2005–2014, Forest Economic Advisors LLC

^c^Average price of Northern Bleached Softwood Kraft (NBSK) delivered to China during 2005–2014, Brian McClay & Associates Inc.

**Table 5:** Cost assumptions for individual strategies for harvested wood products and energy (2014 dollars)

Scenario	Forest region	Sawnwood cost ($/m^3^)^a^	Panel cost ($/m^3^)^b^	Other industrial roundwood cost ($/m^3^)	Pulp cost ($/odt)^c^	Bioenergy cost ($/MWh)
Base case	Northern interior	110	223	110	660	–
	Southern interior	110	223	110	615	
	Coast	110	223	110	645	
Harvest residue for bioenergy	Northern interior	No change				Varies spatially
	Southern interior					
	Coast					
More longer-lived products	Northern interior	No change	2% decrease	No change	2% increase	–
	Southern interior					
	Coast					

^a^Average manufacturing cost for lumber in BC during 2005–2014, Forest Economic Advisors LLC

^b^Average manufacturing cost for OSB (3/8 in.) in Western Canada during 2005–2014, Forest Economic Advisors LLC

^c^Average cost (manufacturing plus transportation) for NBSK in 2012 and 2014, FisherSolve, Fisher International Inc.

**Table 6:** Cost and price assumptions for substituted products in strategies involving displacement effects (2014 dollars)

Scenario	Concrete price ($/tonne)^a^	Steel price ($/tonne)^b^	Fossil fuel energy price ($/MWh)	Concrete cost ($/tonne)^a^	Steel cost ($/tonne)^c^	Fossil fuel energy cost ($/MWh)
Harvest less/restricted harvest/more longer-lived products	65	834	–	64	791	–
Harvest residue for bioenergy	–	–	Varies spatially	–		Varies spatially

^a^NRMCA (2012)

^b^MEPS (2014)

^c^Based on the steel price and the ratio between annual averages of the total revenue and total cost of the steel industry between 2004 and 2012 provided by Statistics Canada (CANSIM Table 301–0006)

Harvests were used to produce generic HWP commodities: sawnwood, other industrial roundwood, panels, and pulp and paper products (Tables 4 and 5). In the more LLP strategy, we assumed a 2% increase in the pulp and paper manufacturing cost and a 2% decrease in the panel production cost due to economies of scale in existing mills. Prices and costs for bioenergy were determined by the LP model for each FMU. Since we assumed there was no bioenergy production from harvest residues in the baseline, no baseline prices and costs were needed. In the bioenergy strategy, the price of bioenergy in each FMU was calculated based on generic electricity price ($120/MWh) and heat price ($8/GJ) weighted by proportions of power and heat that bioenergy generated. The bioenergy production cost in the bioenergy strategy was estimated by the LP model based on the selected facilities and associated production costs (Table 2). The bioenergy production cost also included costs for processing harvest residues and transporting to facilities, as well as the avoided cost of reduced slashburning (Table 7). No cost was assumed for extracting harvest residue from cut blocks to roadside, because the full-tree harvesting approach was assumed to be employed in BC.

**Table 11:** Average annual domestic mitigation and associated economic and socio-economic impacts (in 2016 Canadian dollars), 2017–2050

Strategy	Average annual domestic mitigation^a^ (MtCO_2_e/year)	Average annual domestic mitigation cost ($M/year)	Average domestic mitigation cost per tonne ($/tCO_2_e)	Direct employment impact (full-time equivalent)	Total employment impact (full-time equivalent)	Average annual direct GDP impact ($M/year)	Average annual total GDP impact ($M/year)	Average annual total impact on government revenue ($M/year)
Higher utilization	5.0	−7	−2	0	0	−1	−2	0
Harvest less	1.9	33	21	−431	−909	−45	−91	−6
Bioenergy	4.0	248	79	1143	1862	352	583	65
restricted harvest	2.3	81	36	−1322	−2789	−130	−262	−18
More LLP	1.8	147	97	265	495	−45	−96	−8
Higher utilization + bioenergy	8.5	309	48	1401	2282	299	495	55
Higher utilization + more LLP	6.8	140	25	265	495	−46	−97	−8
Harvest less + more LLP	3.7	179	59	−182	−448	−86	−178	−13
Bioenergy + more LLP	5.2	415	103	1408	2357	307	487	57
Restricted harvest + more LLP	3.5	171	63	−1155	−2475	−159	−323	−23
Higher utilization + bioenergy + More LLP	10.2	457	57	1665	2776	254	399	47
PORT2	12.4	610	43	176	−162	66	52	17
PORT2 w/o restricted harvest	10.8	559	46	1289	2166	184	284	34

^a^Domestic mitigation values here were calculated based on the FMUs with total domestic mitigation greater than 0.01 MtCO_2_e

Economic assumptions for displacement included prices and costs for concrete and steel products and fossil fuel energy. The prices for fossil fuel energy in the bioenergy strategy were the same as the prices for bioenergy, while the unit costs were calculated by dividing the total energy production cost (including fuel cost and production cost) from all fuel sources being substituted by the total bioenergy production (Table 8).

**Table 7:** Cost assumptions for the supply of harvest residues (in 2008 dollars)

Forest region	Processing ($/odt)	Other cost ($/odt)^c^	Avoided cost ($/odt)^d^	Transportation cost		
				Fixed cost ($/odt)^e^	≤50 km ($/odt/km)	>50 km ($/odt/km)
Northern/southern interior	21^a^	8	−5	8	0.31^f^	0.16^f^
Coast	19^b^	8	−5	8	0.36^b^	0.2^b^

^a^Friesen (2013)

^b^MacDonald et al. (2012)

^c^Average of Ralevic et al. (2010), Ralevic (2013), Ryans and Cormier (2009), and Reynolds et al. (2012)

^d^Based on a weighted average cost for slashburning from Baxter (2010)

^e^Average of Ralevic (2013), MacDonald et al. (2012), Gautam et al. (2010), and Kumar et al. (2008)

^f^Ralevic (2013), including grinding costs with pre-piling

### Socio-economic impacts of strategies

The socio-economic impacts of mitigation strategies were analyzed using multipliers from the national input-output (I/O) model (Statistics Canada 2014). The value of a multiplier refers to the increase/decrease in an indicator (e.g., gross domestic product (GDP)) if the demand for the output of a given industry increases/decreases by $1 (or $1 million for employment). In this study, multipliers were used to assess impacts on employment, GDP, and government revenues in BC’s economy in response to changes in the forest sector resulting from the implementation of mitigation strategies. We considered both direct effects and indirect effects on those indicators, where the former refers to the impacts directly induced from a change in an industry’s output, and the latter measures the impacts of further output changes due to interactions among industries within BC in response to the initial changes in the directly affected industry. Given that induced effects may cause double counting (Horne 2008), we did not consider induced effects in our analysis. We also only focused on the socio-economic impacts in response to changes in BC’s forest sector—no impacts resulting from displacement effects in other industries/sectors were estimated.

Five different industries in the forest sector as defined in the North American Industry Classification System (NAICS) (Table 9) were used to estimate socio-economic impacts. Mitigation actions with harvest-related activities were assigned to “forestry and logging”; strategies involving HWP were linked to two manufacturing industries—“wood products manufacture” and “pulp, paper, and paperboard mills”; for the bioenergy strategy, multipliers from the “electric power generation, transmission and distribution” were employed for bioenergy generation, and averages of multipliers for forestry and logging and “truck transportation” were used to represent impacts of harvest residue extraction for bioenergy since this activity is not specified in NAICS. For strategies involving multiple industries, total impacts were estimated as the sum of impacts on all of the relevant industries.

**Table 8:** Cost assumptions for power and heat production using fossil fuels (2008 dollars)

Energy type	Fuel type	Total cost ($/MWh)
Heat	Natural gas	22^a^
	Electricity	42^b^
	Fuel oil	101^c^
	Waste fuels	5^d^
Power	Diesel	259^e^
	Natural gas	35^f^

^a^NEB (2014), Manitoba Hydro (2015), EPA (2013), and IEA (2010)

^b^NEB (2014), Manitoba Hydro (2015), USDC (2011), and Hamilton Home Products (2015)

^c^NRCan (2015), EPA (2013), and IEA (2010)

^d^Waste fuels refer to fuels recovered from industrial processes, such as coke, coke oven gas, petroleum coke, and distilled gas. The total cost was estimated based on EIA (2013b), EPA (2013), and IEA (2010)

^e^NRCan (2015), Dunn (2011), and Osler (2011)

^f^NEB (2014), Manitoba Hydro (2015), and EIA (2013a)

Our analysis estimated the direct and indirect effects using multipliers shown in Table 9. For each mitigation strategy, the initial change in an industry was estimated as the change in total revenues of that industry between the baseline and a mitigation scenario. Following the initial change, total direct/indirect socio-economic impacts on GDP (*SE*_GDP_) for the entire period (2017–2050) were estimated using the corresponding multipliers:

$$ {SE}_{\mathrm{GDP}}=\sum_j^J\sum_t^T\frac{GDP_{j t}}{{\left(1+ r\right)}^t}=\sum_j^J\sum_t^T\frac{\Delta { T R}_{j t}\times {m}_j^{GDP}}{{\left(1+ r\right)}^t} $$

where *GDP*_*jt*_ is the GDP impact from industry *j* at time *t*, Δ*TR*_*jt*_ is the total revenue change estimated in MEA-FCM and ${m}_j^{GDP}$ is the multiplier for direct/indirect GDP impacts of industry *i*, and *r* is the discount rate.

Because there are no multipliers specified for impacts on government revenue in the national I/O model, we used the sum of the multipliers for the government revenue-related components that are used for GDP calculations (personal and business income taxes were not included). We did not separately estimate impacts on provincial government revenue in BC since no associated multipliers were available; rather, we estimated the impact on total government revenue for all federal, provincial, and municipal governments:

$$ {SE}_{\mathrm{revenue}}=\sum_j^J\sum_t^T\frac{GR_{j t}}{{\left(1+ r\right)}^t}=\sum_j^J\sum_t^T\frac{\Delta { T R}_{j t}\times \left({m}_j^{T p}+{m}_j^{T n}-{m}_j^{Sp}-{m}_j^{Sn}\right)}{{\left(1+ r\right)}^t} $$

where *GR*_*jt*_ is the total government revenue impact from industry *j* at time *t*; ${m}_j^{Tp}$ and ${m}_j^{Tn}$ are multipliers for taxes on products and production of industry *j*, respectively; and ${m}_j^{Sp}$ and ${m}_j^{Sn}$ are multipliers for subsidies on products and production of industry *j*, respectively.

The direct impacts on employment were not examined using employment multipliers because most mitigation actions involved in the strategies were so specific that the available multipliers were too general and cannot reflect their impacts on employment appropriately. Instead, we estimated the direct impacts on jobs (${SE}_{\mathrm{direct}}^{\mathrm{Job}}$) by multiplying a labor intensity parameter (e.g., person-year (PY)/m^3^, see Table 10) for each industry with the corresponding biophysical change that occurred in that industry when a given mitigation strategy was implemented:

**Table 9:** Industries identified for the forest (including bioenergy) sector and associated multipliers

Industry (NAICS code)	Direct jobs	Indirect jobs	DirectGDP	Indirect GDP	Total govt. revenue
Forestry and logging (113)	3.74	3.21	0.38	0.29	0.05
Harvest residue extraction^a^	4.84	2.89	–	–	–
Wood products manufacture (3212)	3.30	4.06	0.31	0.38	0.04
Pulp, paper, and paperboard mills (3221)	1.84	3.07	0.27	0.32	0.04
Electric power generation, transmission and distribution (2211)	2.46	1.77	0.81	0.11	0.05

^a^This industry is not specified in NAICS and was artificially made by using averages of the multipliers for “Forestry and Logging” and “Truck Transportation” industries. Only impacts of harvest residue extraction on employment were considered in the socio-economic impacts of the Bioenergy strategy, because costs for extracting harvest residues were included in bioenergy generation, as were the associated socio-economic impacts

$$ {SE}_{\mathrm{direct}}^{\mathrm{Job}}=\sum_j^J\sum_t^T{PY}_{j t}=\sum_j^J\sum_t^T\Delta {Q}_{j t}\times {k}_j $$

where *PY*_*jt*_ refers to the direct change in person-year (the amount of work done by one person in a year) required in industry *j* at time *t*, Δ*Q*_*jt*_ represents the biophysical changes (e.g., harvest volume, volume of harvest residue for bioenergy, or HWP or bioenergy production) due to strategy implementation, and *k*_*j*_ is the labor intensity parameter for industry *j*.

Specifically, the labor intensity required for every cubic meter of fiber harvested or manufactured was estimated for each of the forestry and logging, wood products manufacture, and pulp, paper, and paperboard mills’ industries by dividing the annual average of the total number of employees in each industry by the annual average of total industrial roundwood harvest in BC during 2009–2013. The data were derived from Statistics Canada (CANSIM 2015) and the National Forestry Database (NFD 2015), respectively. We used estimates in FPInnovations (2010) as the labor intensity for harvest residue extraction, and the labor requirement for an 8 MWe combined heat and power (CHP) steam turbine (FPAC and FPInnovations 2011) to estimate the labor intensity for bioenergy generation.

Although we did not use multipliers to calculate the direct effects on employment, we assumed that the ratio between multipliers for direct and indirect effects on employment is accurate. We then used the ratio and the estimated direct effects described previously to calculate the indirect impacts on jobs (${SE}_{\mathrm{indirect}}^{\mathrm{Job}}$):

$$ {SE}_{\mathrm{indirect}}^{\mathrm{Job}}=\sum_j^J\sum_t^T{PY}_{j t}^{\hbox{'}}=\sum_j^J\sum_t^T{PY}_{j t}\times \frac{m_j^{\hbox{'}}}{m_j} $$

where ${PY}_{jt}^{\hbox{'}}$ is the indirect change in PY required in industry *j* at time *t* and *m*_*j*_and ${m}_j^{\hbox{'}}$are multipliers for direct and indirect effects on employment, respectively.

## Results

### Global mitigation impacts

**Fig. 3:**
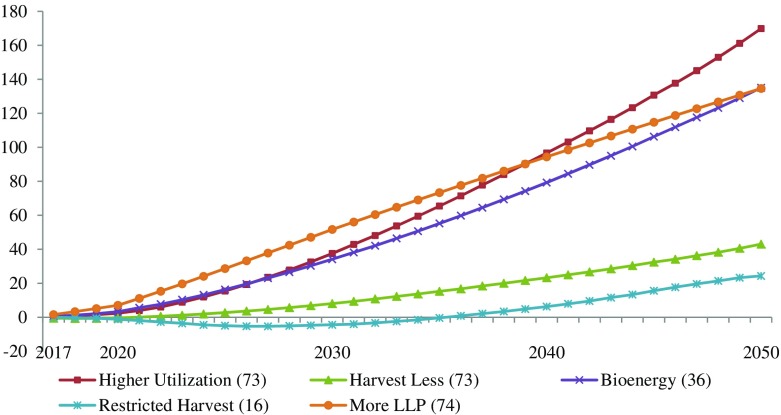
Cumulative global mitigation impacts of individual strategies, 2017–2050, relative to the baseline. Values in the *brackets* are the numbers of FMUs with global mitigation impacts greater than 0.01 MtCO_2_e–only these FMUs are included in the impacts shown

**Fig. 4:**
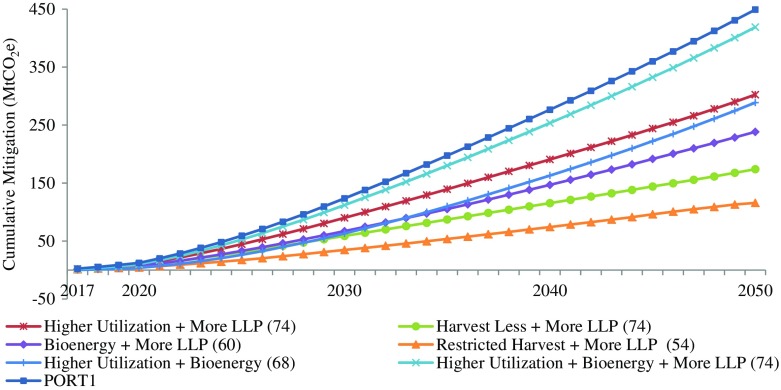
Global mitigation impacts of combined strategies and the portfolio that maximizes global mitigation (PORT1), 2017–2050. Values in the *brackets* are the numbers of FMUs with global mitigation impacts greater than 0.01 MtCO2e—only these FMUs are included in the impacts shown

**Fig. 5:**
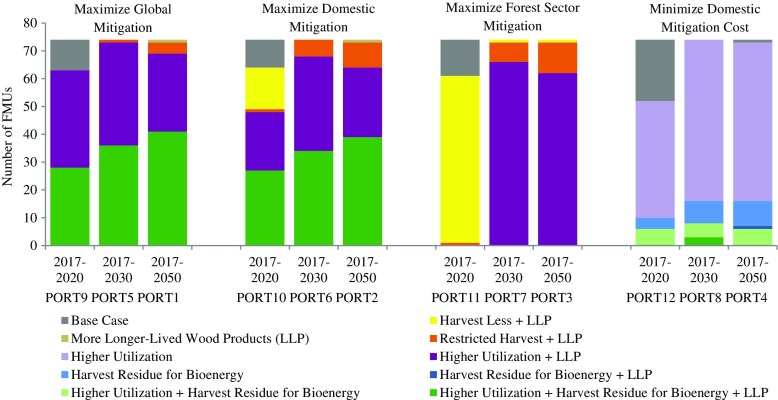
Distribution of the strategy mix in portfolios

The biophysical impacts of mitigation strategies and portfolios are shown as cumulative global mitigation during the entire time period in Figs. 3 and 4. The initial impacts of the restricted harvest strategy (2017–2035) and the harvest less strategy (2017–2021) were negative (i.e., the strategies did not achieve positive cumulative mitigation until a number of years after initial implementation). Cumulative mitigation in all individual strategies changed nonlinearly over time, except the more LLP strategy (which was linear with two different slopes before and after 2020 due to the initial ramp-up period), and the magnitudes of the cumulative mitigation in 2050 ranged from a low of 24.3 MtCO_2_e (restricted harvest) to a high of 169.9 MtCO_2_e (higher utilization) (Fig. 3). The cumulative global mitigation resulting from strategy combinations and portfolios was significantly higher, at 449.1 MtCO_2_e for PORT1 (Fig. 4) with a regionally differentiated strategy mix (Fig. 5). This is about 38% of the estimate of 1180 MtCO_2_e for global mitigation resulting from strategies implemented across Canada provided by Smyth et al. (2014).

### Economic and socio-economic impacts

We summarize average annual domestic mitigation and associated costs, as well as socio-economic impacts, over the whole period for all strategies and PORT2 in Table 11. In terms of individual strategies, the higher utilization strategy had the highest annual average domestic mitigation (5.0 MtCO_2_e/year) and the lowest mitigation cost but it had minimal socio-economic impacts. The bioenergy strategy had the second highest mitigation (4.0 MtCO_2_e/year) and the greatest socio-economic contributions, although the annual mitigation cost was also the highest ($248 M/year). The more LLP strategy resulted in the least domestic mitigation (1.8 MtCO_2_e/year) with the highest cost per tonne value ($97/tCO_2_e). For combined strategies, “higher utilization” plus “bioenergy” plus “more LLP” had the highest average domestic mitigation of 10.2 MtCO_2_e/year with a cost of $57/tCO_2_e. It also considerably increased socio-economic benefits. higher utilization plus bioenergy achieved the second highest domestic mitigation of 8.5 MtCO_2_e/year and had positive socio-economic impacts, especially for GDP and government revenue. Since the restricted harvest strategy provided relatively little domestic mitigation but had significant negative socio-economic impacts, we included two versions of PORT2 (with/without the restricted harvest strategy) for comparison (Table 11). Clearly, both versions of PORT2 generated large mitigation impacts with relatively low cost per tonne values (with little of the mitigation arising from the restricted harvest strategy), but there were considerable socio-economic benefits in the version without the restricted harvest strategy.

**Table 10:** Industry distribution among strategies and labor intensity assumptions

Strategy	Logging (person-year/million m^3^)	Harvest residue extraction (person-year/million m^3^)	Wood manufacture (person-year/million m^3^)	Pulp and paper manufacture (person-year/million odt)	Bioenergy generation (person-year/million m^3^)
Higher utilization	170				
Harvest less/restricted harvest	170		390	270	
Bioenergy		90			30
More LLP			390	270	
Higher utilization + bioenergy	170	90			30
Higher utilization + more LLP	170		390	270	
Harvest less + more LLP/restricted harvest + more LLP	170		390	270	
Bioenergy + more LLP		90	390	270	30
Higher utilization + bioenergy + more LLP	170	90	390	270	30

**Fig. 6:**
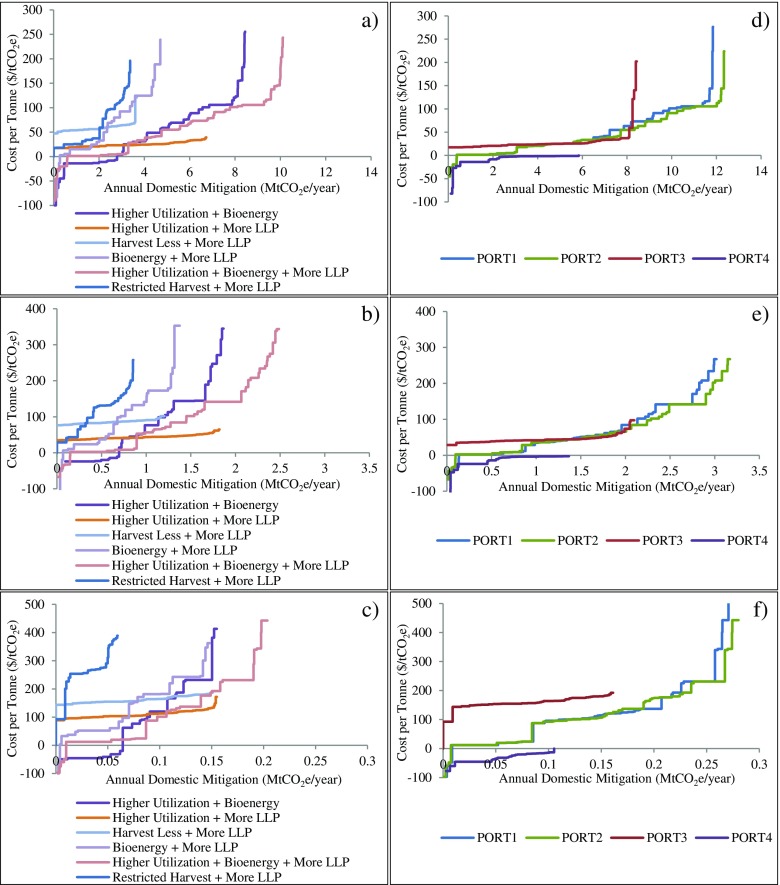
Cost curves for strategies and portfolios in various time periods. The *left* three panels are cost curves for combined strategies during 2017–2050 (**a**), 2017–2030 (**b**), and 2017–2020 (**c**); the *right* three panels are cost curves for domestic mitigation for the four portfolios during 2017–2050 (**d**), 2017–2030 (**e**), and 2017–2020 (**f**). Some extreme values have been eliminated for display purposes

**Fig. 7:**
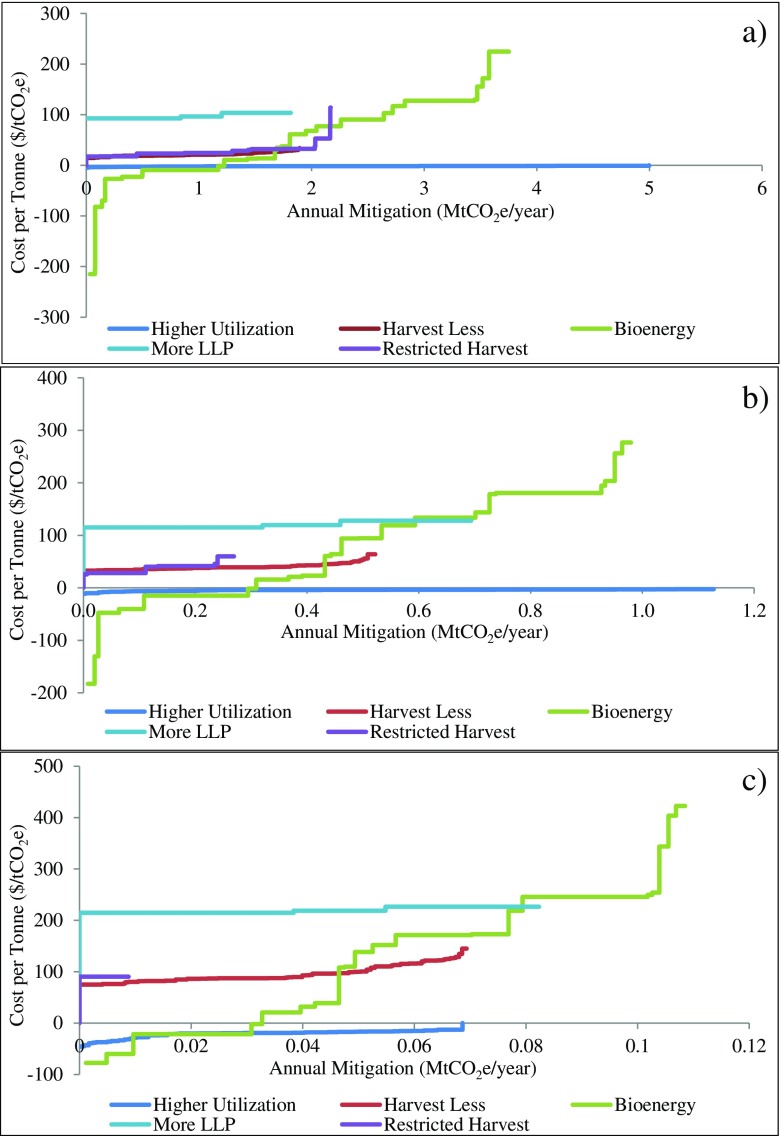
Cost curves for individual strategies in **a** long term (2017–2050), **b** mid term (2017–2030), and **c** short term (2017–2020). Some extreme values were eliminated for display purposes

Cost curves showing the spatial variation of cost per tonne values across FMUs for combined strategies and portfolios are provided in Fig. 6 (see Fig. 7 for individual strategies). They were constructed by ranking cost per tonne values for all FMUs from the lowest to the highest and plotting against cumulative annual domestic mitigation. Because neither cost per tonne nor annual mitigation were continuous across FMUs, cost curves were discrete with each horizontal line segment representing the annual domestic mitigation in a single FMU at the estimated cost. For each strategy, the curve can be used to estimate the implementation costs of a strategy if specific annual mitigation targets need to be met in the period or to estimate how much mitigation can be achieved by a strategy with a given budget. By comparing cost curves across strategies, we can interpret the cost-effectiveness of a strategy relative to others at a certain mitigation level. For example, in panel a of Fig. 6, higher utilization plus bioenergy was more cost-effective than higher utilization plus bioenergy plus more LLP if the annual domestic mitigation was less than 3 MtCO_2_e/year, but the former became less cost-effective if more mitigation was required. Significant increases in mitigation potential and decreases in cost per tonne can be seen in Fig. 6 if longer time periods were considered (panels a, b, and c). On the right panels, the cumulative annual domestic mitigation and cost levels were similar when FMUs were selected to maximize global and domestic mitigation (PORT1 and PORT2). However, only two thirds of the amount could be achieved if the goal was to maximize mitigation in the forest sector (PORT3), and the mitigation was more expensive. Even less (nearly half of the total level achieved by PORT2) can be achieved with net economic gains when domestic mitigation cost was minimized (PORT4). More mitigation can be accomplished at a lower cost level in PORT1 and PORT2 than any strategies during the same time period (e.g., panels a and d). Finally, a portfolio that maximizes global mitigation (PORT1) achieves slightly less domestic mitigation than a portfolio that focuses on maximizing domestic mitigation (PORT2).

To examine the economic potential of mitigation relevant to BC’s emission reduction targets, we calculated the annual averages of domestic mitigation in PORT2 with and without the restricted harvest strategy at various cost levels in Tables 12 and 13, respectively. We found that, in both cases, the economic potential of mitigation impacts increased substantially in later decades or if higher costs were tolerated, which was consistent with the nonlinear curves in Figs. 3 and 4. However, significant reductions in the forest sector mitigation were found when restricted harvest plus more LLP was removed from the portfolio (Table 13), indicating that excluding such a strategy reduced mitigation potential in the forest sector if no displacement effects were considered. BC’s emissions targets are a 33% reduction in 2020 and an 80% reduction in 2050 relative to the 2007 level (Government of British Columbia 2007). Under PORT2, including the restricted harvest strategy, we calculated that 1 MtCO_2_e would be mitigated under $100/tCO_2_e in 2017–2020, which is only about 1.5% of the province’s 2007 emissions of 66 MtCO_2_e based on BC’s latest GHG inventory (Government of British Columbia 2016), while the contribution of PORT2 under $100/tCO_2_e in 2050 is 18.2 MtCO_2_e, equivalent to 28% of 2007 emissions (or 35% of the 2050 target).

**Table 12:** Annual averages of mitigation potentials in PORT2 at various cost levels for different periods, MtCO_2_e/year

Period	Scale	<$30/tCO_2_e	<$50/tCO_2_e	<$100/tCO_2_e	All costs
2017–2020	Forest sector	−0.6	−0.6	−0.4	−0.8
	Domestic	0.7	0.7	1.0	2.5
2021–2030	Forest sector	0.5	2.4	3.3	3.1
	Domestic	2.9	5.0	7.4	9.8
2031–2040	Forest sector	5.4	6.9	7.1	8.2
	Domestic	7.9	10.8	12.7	14.4
2041–2050	Forest sector	6.3	7.5	9.6	10.3
	Domestic	8.5	10.1	15.2	16.8
2017–2050	Forest sector	3.5	4.9	5.8	6.2
	Domestic	5.8	7.7	10.5	12.4

**Table 13:** Annual averages of mitigation potentials in PORT2 without the “Restricted Harvest” strategy at various cost levels, by period

Period	Scale	<$30/tCO_2_e	<$50/tCO_2_e	<$100/tCO_2_e	All costs
2017–2020	Forest sector	−0.6	−0.6	−0.5	−1.0
	Domestic	0.7	0.7	1.0	2.4
2021–2030	Forest sector	−0.1	1.3	1.4	1.3
	Domestic	2.7	4.4	6.5	9.0
2031–2040	Forest sector	3.4	3.4	3.8	4.9
	Domestic	6.5	8.6	10.7	12.4
2041–2050	Forest sector	4.0	3.6	5.5	6.4
	Domestic	7.1	7.9	12.6	14.6
2017–2050	Forest sector	2.1	2.4	3.1	3.6
	Domestic	4.9	6.2	8.9	10.8

## Discussion

Our results indicated that implementing two or more strategies simultaneously in a FMU would achieve more mitigation than having only one individual strategy. However, combined strategies may not necessarily be more cost-effective or have more socio-economic benefits. For example, the combination of the bioenergy strategy and the more LLP strategy increased the average domestic mitigation from 4.0 MtCO_2_e in the bioenergy strategy alone to 5.2 MtCO_2_e in the long term but resulted in increased mitigation cost and less GDP and government revenue (Table 11). Strategies may or may not affect each other when combined (i.e., they are additive or not). In particular, the higher utilization strategy and the bioenergy strategy were not additive because higher utilization in harvest caused fewer harvest residues left on site, which resulted in a smaller supply for local bioenergy. In contrast, the more LLP strategy and the bioenergy strategy were additive, as shifting the wood product mix for a given harvest volume did not affect the amount of harvest residues. Therefore, whether a combined strategy was preferred varied, depending on the interaction between mitigation actions and policy goals. Further, our results also suggested that a portfolio would be superior to any single strategy applied across all FMUs. For instance, if the goal is to maximize domestic mitigation in the long run, PORT2 (panel d, Fig. 6) achieved more domestic mitigation than any individual strategy during the same period (panel a, Fig. 6). Finally, similar to what was found by Smyth et al. (2014), there was a trade-off in portfolios between long-term mitigation and short-term mitigation—less mitigation would be achieved in the long term to 2050 if the best short-term portfolio for 2020 was applied. This is because most of the strategies’ mitigation contributions are nonlinear over time (Fig. 3), and different strategies would be selected in the portfolios depending on the time scale of mitigation goals.

The higher utilization strategy assumed that the same amount of wood was harvested in a smaller area with a higher utilization level and that the same product mix could be achieved, i.e., incremental harvest from a stand had the same quality as the regular harvest from the stand, although this may not be the case in practice as the incremental harvest could be of lower quality and produce less valuable wood products. However, changes in the shares among different harvest products (softwood, hardwood, salvage harvest, and harvest residues) were captured in our analysis. This strategy resulted in the highest cumulative mitigation, but most mitigation was achieved in later years (Fig. 3) because fewer residues were left to decay and more carbon was sequestrated in unharvested stands, relative to the baseline. This strategy had a very flat cost curve that was close to the horizontal axis because unchanged harvest levels and a small logging cost reduction caused a small increase in total net revenue. The unchanged harvest level also meant that no new jobs were created and that there was insignificant contribution to other socio-economic indicators.

The harvest less strategy in our analysis assumed a reduced harvest level and increased costs because more dispersed cut blocks were needed to keep the same harvest characteristics (e.g., diameters, tree species, etc.). The mitigation impact of this strategy was limited because reduced emissions and increased carbon density resulting from harvesting less were offset over time by a lower carbon uptake rate due to less post-harvest regeneration and negative displacement effects since more emission-intensive products (e.g., concrete and steel) were assumed to be used to meet society’s demands. Also, because of higher logging costs and lower log production, respectively, less net profit was generated and negative socio-economic impacts were found. Therefore, this strategy highlights trade-offs between carbon density and carbon uptake rate based on the society’s demand for biomass (Lemprière et al. 2013), even though it had higher forest sector mitigation potential in the short term (Fig. 5).

In the bioenergy scenario, how much mitigation could be achieved at what cost varied greatly across FMUs because different numbers and types of bioenergy facilities were selected to substitute different fossil fuels by the LP model based on local energy demands, harvest residue availability within each FMU, transportation distances (simplified), and production costs for both bioenergy and fossil fuel energy being displaced. In this study, we assumed that in most FMUs, nearly 80% of the electricity was hydro-electricity and about 35% of heat was produced from biomass, which had no displacement benefits from bioenergy. Most mitigation benefits came from substituting bioenergy for heat and power generated using natural gas and fuel oil/diesel, and the percentage of these fossil fuels in total energy consumption varied across FMUs. Consequently, only 36 FMUs showed positive mitigation in this strategy. Among those, considerable cumulative mitigation was estimated in some areas and significant variation in cost per tonne was also observed (panel a, Fig. 7). High mitigation from this strategy was mostly from FMUs with large populations due to higher energy demands, but the strategy also incurred relatively high costs because in populated areas, a large proportion of fossil fuel energy is from natural gas which is generally much cheaper than bioenergy. In some other FMUs, especially those in the northern interior of BC, some remote communities are not connected to the power grid and produce energy using diesel generators with higher costs, indicating that lower energy costs would be possible in those FMUs if they converted to bioenergy, if the transportation costs for harvest residues were competitive. However, we did not directly estimate these effects.

Because there was no bioenergy from harvest residues assumed in the baseline and no socio-economic impacts were estimated for displaced energy facilities, harvest residue-based bioenergy production was a new industry in this mitigation scenario and thus substantial revenue was created, generating large socio-economic benefits.

**Fig. 8:**
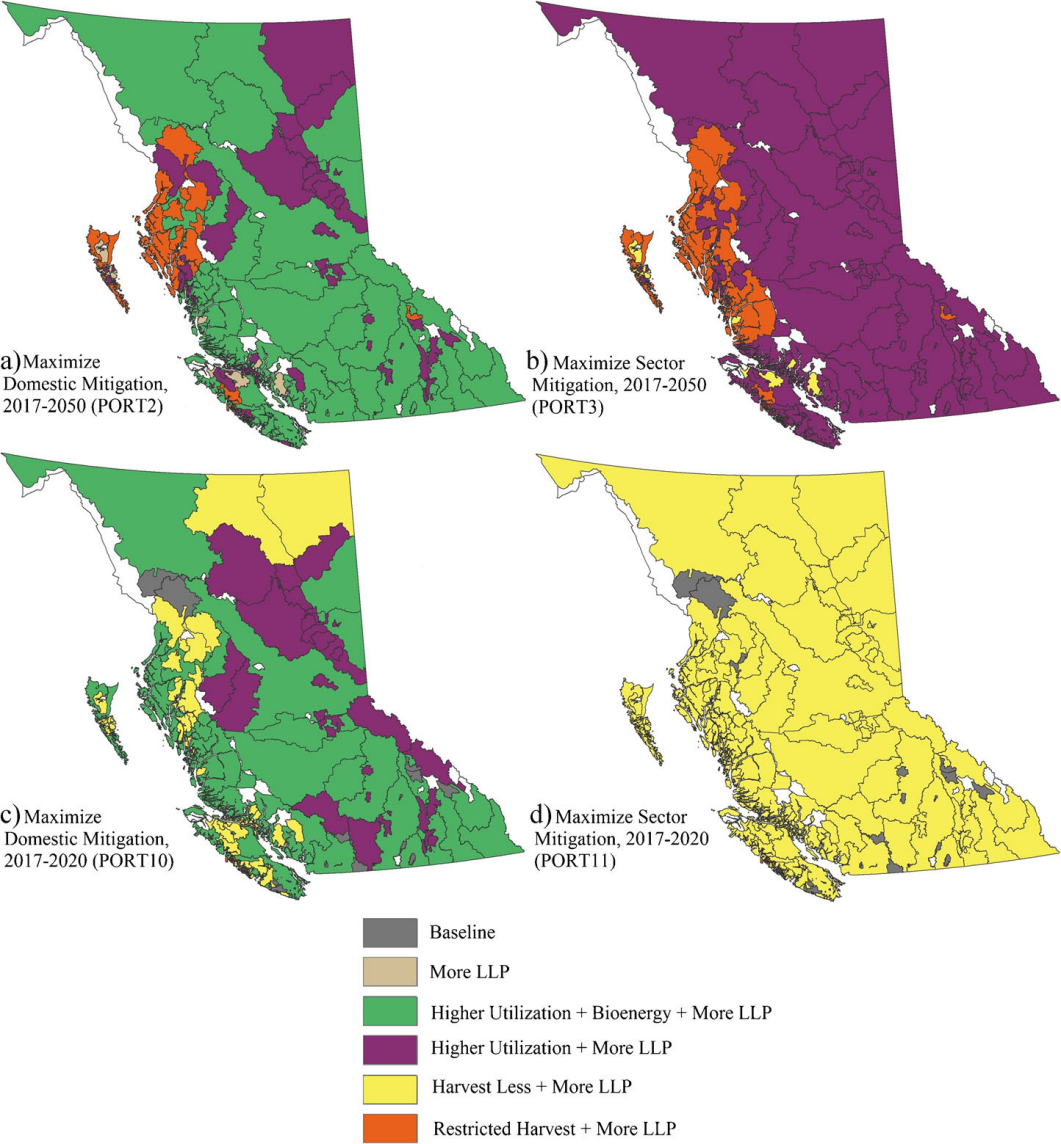
Spatial distribution of the strategy mix in portfolios with short-term (2017–2020) and long-term (2017–2050) goals. FMUs *without color* refer to spatial units that were not included in this study. Different strategy mixes were selected for the goal of maximizing forest sector mitigation versus the goal of maximizing domestic mitigation. For forest sector mitigation (**b**, **d**), Higher Utilization + More LLP and Harvest Less + More LLP were the dominant strategies in the long term and short term, respectively, because of their significant mitigation potentials in the forest sector. When domestic displacement effects were considered (**a**, **c**), the Bioenergy strategy was included in the best strategies in many FMUs except areas where large amounts of harvest residues were available, but local heat and electricity demand was relatively low. Compared to ( **a**), Harvest Less + More LLP was selected in ( **c**) in some coastal areas because large proportion of the harvest in those areas are from mature or old-growth forest stands with a high carbon density; therefore, less harvest provided greater gains in the carbon stock initially. In ( **b**), Higher Utilization + More LLP was the best for the interior of the province because all mitigation from this strategy was gained within the forest sector, and the Restricted Harvest strategy was preferred for the coast because of the great proportion of old-growth harvest there. In ( **d**), the Harvest Less + More LLP strategy almost completely replaced other strategies in all FMUs because in the short term a reduction in the harvest level always resulted in the highest initial forest mitigation

The restricted harvest strategy resulted in two outcomes compared to baseline harvest. In about three quarters of FMUs, restricting harvest to stands less than 250 years old while not also lowering the harvest target forced the model to harvest larger areas within a FMU to achieve the baseline harvest level because volume per hectare harvested is greater in older stands. In the remaining quarter of FMUs, however, the model was unable to find a sufficient amount of stands eligible for harvest within the FMU and the harvest targets were not achieved. Over time, the total area of harvest declined, resulting in decreasing total revenue in the forest sector and negative socio-economic impacts. Given that there are few stands over 250 years in BC’s interior region, harvest levels could be maintained in most FMUs by shifting harvest to younger stands based on the model assumptions. Negative mitigation impacts were found in those FMUs even if only the forest sector mitigation was considered (no displacement effects), indicating that the additional carbon preserved in old stands would be offset by the increased carbon losses resulting from increased harvest of younger stands. Without considering socio-economic impacts and values for other ecosystem services, most mitigation benefits generated by this strategy would be limited to the central and north coast of BC (panels a and b, Fig. 8) where harvest levels were significantly lowered because old growth represents a greater proportion of the baseline harvest in those areas and there are insufficient younger stands to meet harvest targets. In fact, those areas covered most of the Great Bear Rainforest protection area where ecosystem-based management is implemented and logging is largely prohibited (BCMoFLNRO 2016).

Note that the restricted harvest strategy was developed based on simplified assumptions and do not reflect actual harvest practices and policies currently employed in BC (e.g., a stand age of 250 years as a criterion for restricting harvesting of old-growth forest would be too high for the interior area, and an average utilization rate of 85% may overestimate utilization in coastal old-growth stands). Furthermore, BC’s government policy on removing any land from what is harvestable results in an a priori reduction in the allowed harvestable volume, so the harvest rate on younger stands would remain the same. Further research is needed to refine utilization levels for different logging practices (e.g., helicopter logging that can be used in BC coastal forests typically has lower utilization standards and higher costs) to review and, where necessary, update yield curves and estimates of carbon sequestration in old-growth stands and to refine assumptions about compensation costs and changes in harvest targets that may be associated with the implementation of an age-restricted harvest strategy. Thus, we caution that this analysis of a restricted harvest strategy should not be used for policy development in the province without further assessment.

The only strategy regarding wood use in this study, the more LLP strategy, had linear cumulative mitigation over time because a constant 4% shift was assumed in the annual production from pulp and paper products to panels. This strategy greatly increased the carbon storage in HWP by transferring carbon from pulp and paper products (2 years half-life) to panels (25 years half-life) (IPCC 2006). In addition, more panel products further enhanced the mitigation impact via displacement effects. Consequently, this strategy demonstrated the highest cumulative global mitigation until 2040 when it was surpassed by the higher utilization strategy. Because of the fixed shift in product commodities, this strategy had a flat cost curve (Fig. 7) with only three different costs per tonne values recognizing the different cost and price assumptions we used for the three forest regions in the province. The cost per tonne values was high, because the pulp and paper industry is relatively more capital intensive—pulp and paper mills usually have larger proportion of capital investments in equipment and facilities than sawmills. Therefore, an equal proportional change in the production would cause more revenue loss in pulp and paper industry than would be gained in the panel industry. This is also the reason why shifting total production to panels would cause small negative GDP and government revenue effects (Table 11).

Although the strategy mixes selected by FMU across portfolios varied, higher utilization plus more LLP and higher utilization plus bioenergy plus more LLP were chosen in most FMUs in portfolios considering displacement effects (Fig. 5), indicating that those two strategies are among the best examined here when considering mitigation efforts of BC’s forest sector from a systems perspective. For the forest sector alone (ignoring displacement effects), however, the bioenergy strategy was not selected in portfolios for either long-term or short-term mitigation goals, because burning harvest residues for bioenergy created limited mitigation benefits relative to other strategies (panel b, Fig. 8). When the goal was to minimize domestic mitigation cost regardless of GHG emissions reductions, completely different strategies were chosen for the portfolios.

**Fig. 9:**
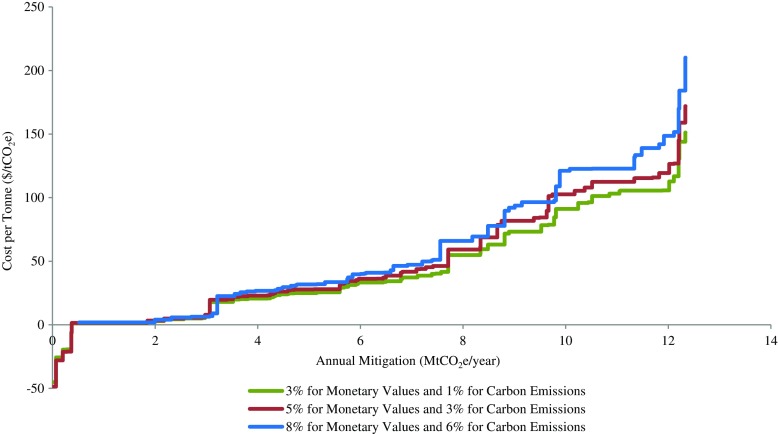
Sensitivity analysis of the cost of the portfolio that maximizes domestic mitigation during 2017–2050 (PORT2) based on three different assumptions about discount rates

A sensitivity analysis of discount rates was conducted for PORT2 to examine possible changes in cost per tonne values if higher monetary and carbon discount rates were applied. Considering 3 and 1% discount rates to be the most conservative assumptions, we re-estimated the cost curve for PORT2 based on a moderate assumption of 5 and 3 and also a high assumption of 8 and 6%, respectively (Fig. 9). The results suggested that the moderate assumption would increase the cost curve by up to 13% (from $106/tCO_2_e to $119/tCO_2_e) to achieve up to 12 MtCO_2_e per year, and the ambitious assumption would shift the curve upwards by up to 25% (from $106/tCO_2_e to $149/tCO_2_e) to achieve the same annual mitigation.

The biophysical mitigation potential in this study is by no means the upper limit of what can be achieved if society is ambitious in implementing changes to reduce GHG emissions. Other mitigation options involving forests have not been assessed here, such as reductions in deforestation rates, rehabilitation of stands affected by natural disturbances that have not successfully regenerated, afforestation including short-rotation bioenergy plantations (Amichev et al. 2012), and intensive forest management including nutrient management (Lemprière et al. 2013). Potential impacts of climate change on mitigation potentials were not considered here but need to be addressed in ongoing research. For the economic and socio-economic analyses, future work could consider dynamic price and cost assumptions.

## Conclusions

In this study, we examined how much BC’s forest sector may contribute to climate change mitigation by developing several potential mitigation strategies and portfolios and examining their biophysical impacts, costs, and socio-economic impacts on BC’s economy. The results indicated that significant mitigation with positive socio-economic benefits would be possible if long-term regionally differentiated strategies were implemented soon. Our analysis estimated that regionally differentiated portfolios provided the highest cumulative global mitigation (PORT1) and the highest cumulative domestic mitigation (PORT2) by 2050 with similar strategy mixes that consisted of combinations of the higher utilization, bioenergy, restricted harvest, and more LLP strategies. By implementing PORT2 starting in 2017, BC’s forest sector could contribute 35% of the province’s emission reduction target in 2050 at a cost of less than $100/tCO_2_e, although implementation of such a forest sector mitigation portfolio would also cost $610 million Canadian dollars every year on average between 2017 and 2050.

Consideration of mitigation strategies would need to balance the multiple objectives that exist for BC’s forests and forest sector. This study is the first regionally differentiated forest-related mitigation study for the entire province of BC, incorporating biophysical, economic, and socio-economic impacts using rigorous quantitative analyses from a systems perspective. Moreover, our findings indicate that mitigation benefits from strategy implementation in one country could be extended to other countries via international trade if exported wood products are used to displace emission-intensive materials.

Activities related to higher utilization and increased use of longer-lived wood products and bioenergy from waste wood have been recognized in this study and in several other countries (Werner et al. 2010; Lundmark et al. 2014; Nordström et al. 2016; FAO 2016) as effective options to mitigate climate change. Based on these analyses, we recommend further exploration of potential forest sector-based mitigation activities globally. The analyses conducted in this study contribute to the global understanding of forest sector mitigation options by clearly outlining economic aspects of the mitigation under various goals and over various time periods and by synthesizing the methods, tools, and datasets needed to quantify mitigation activities. An understanding of economically feasible and socio-economically attractive mitigation strategies or portfolios helps decision makers with long-term planning for land sector contributions to GHG emission reduction efforts, and with efforts to obtain the social license to implement mitigation strategies, which will require dialogue with various stakeholders.

## Appendix

### Modeling mitigation strategy impacts and mitigation implications on BC’s forest sector

The greenhouse gas (GHG) emissions and removals in the forest ecosystem resulting from mitigation strategies described in this study were estimated using the Carbon Budget Model for the Canadian Forest Sector (CBM-CFS3). This model is discussed in detail by Kurz et al. (2009). It was originally developed in the late 1980s (Kurz et al. 2002) and now forms one of the core modeling engines of the National Forest Carbon Monitoring, Accounting and Reporting System in Canada. CBM-CFS3 simulates annual carbon transfers that are associated with ecosystem processes and natural and anthropogenic disturbances (e.g., wildfire and harvesting) between the atmosphere and 10 biomass pools and 11 dead organic matter pools in the forest ecosystem. The model integrates forest inventory data, growth and yield data, and information on forest management practices and natural disturbance impacts. For this study, the forest inventory data were mainly from BC Ministry of Forests, Lands, and Natural Resource Operations. The mitigation impacts on the forest ecosystem were then modeled by comparing changes in the carbon pools between the baseline scenario and the scenario with implementation of mitigation strategies.

The carbon that was transferred out of the forest ecosystem due to harvest was then modeled by the Carbon Budget Model Framework for Harvested Wood Products (CBM-FHWP)—an analytical tool that tracks the fate of harvested carbon throughout the lifetime of harvest wood products (HWP) including bioenergy and includes post-consumer treatment. CBM-FHWP tracks the carbon flow associated with HWPs all over the world as long as they are originally harvested in Canada. This framework is part of Canada’s annual GHG National Inventory Report. The model uses production and export data for wood commodities from the UN Food and Agriculture Organization. The framework modeled HWP carbon with the assumption that sawnwood and other industrial roundwood have a 35-year half-life and that panels and pulp and paper have half-lives of 25 and 2 years, respectively (IPCC 2013). Bioenergy emissions resulting from discarded products were included in the framework by assuming that 10% of discarded solid wood and paper products were used for energy and the rest went to landfills with instant oxidation.

The baseline scenario assumed clear-cutting harvest with 85% of merchantable stem biomass transferred to HWP throughout the province. A few years after harvest, residues on half of the harvest area were piled and burned and residues on the other half area were left in the stands for natural decay—no harvest residues were used for any form of HWP in the baseline. All regular harvest has regeneration obligations in order to maintain sustainability. Salvage harvest was applied in post-fire or mountain pine beetle damaged stands and contributed 6% of the total harvested wood. HWPs in the baseline were categorized as sawnwood, panels, other solid wood, and pulp and paper with a constant commodity mix in the future. Mitigation strategies described in this study included four related to forest management, one for HWP, and six different combinations of the five individual strategies.

The higher utilization strategy increased the average utilization rate for harvest from 85 to 86.5% during 2017–2020 as a ramp-up period and further to 90% during 2021–2050. The proportion of salvage harvest was also increased by 4%. This strategy resulted in more harvest per hectare while keeping the total harvest area unchanged. This strategy was expected to reduce harvest residues and remove more damaged timber, thus lowering carbon emissions from slashburning and natural decay. In addition, harvest areas became smaller, thus increasing the net carbon removals in the forest ecosystem.

The harvest less strategy reduced the total harvest volume by 2% in all forest management units. The direct impact of this strategy was decrease in production of all wood products. We assumed that cut blocks became more dispersed in order to maintain the quality of harvested wood. This strategy reduced the total amount of carbon transferred out of the ecosystem by reducing the harvest volume, but fewer wood products meant there was a requirement for more emission-intensive materials as substitute.

The harvest residue for bioenergy strategy kept the harvest level and utilization rate in the baseline scenario unchanged but reduced the amount of slashburning and a portion (10% for the ramp-up period and 25% for the remaining years) of harvest residues (including branches, small trees, and snags) that would have been either burned or left on site were collected and transported to up to nine different types of hypothetical bioenergy facilities to produce power and heat in place of fossil fuel energies (coal, natural gas, diesel/fuel oil, etc.) within the forest management unit. This strategy not only reduced GHG emissions from slashburning and fossil fuel burning for energy production but also increased GHG emissions from biomass transportation, processing, and combustion. In general, for the same amount of wood, burning in a controlled environment for energy generation would lead to less GHG emissions than slashburning at roadside, because slashburning creates a large amount of methane due to incomplete burning. However, since different measurements were used for the amount of slashburning (percentage of harvest area) and the amount of harvest residue for bioenergy (percentage of harvest residue carbon), more wood was burned for energy than what was avoided from slashburning and, therefore, the mitigation benefit of this strategy greatly relied on displacement effects—avoided emission from fossil fuels being substituted.

The restricted harvest strategy limited harvest to stands less than 250 years old during 2017–2050. Old forests typically store more carbon than managed forests because they have higher carbon density and thus would take longer time to restore the carbon if they are harvested. Given the age structure of BC’s forests, this strategy was expected to mainly constrain old-growth harvest in coastal regions where old forests occupy a much higher proportion of the forest than in the interior, and local ecosystems and wildlife habitats greatly rely on old-growth forests. Restricting old-growth harvest would lower the carbon loss from harvesting old trees but may also increase harvesting in younger stands, thereby offsetting some portion of the carbon benefits since younger stands that are harvested would have had a higher capacity to sequestrate carbon from the atmosphere if left to grow. In coastal areas with less available young stands, harvest volumes would drop and carbon benefits may be gained depending on the magnitude of the displacement effect resulting from less harvest.

The more longer-lived product strategy was a mitigation option that focused on HWP rather than forest management. This strategy assumed that, with the same harvest level as in the baseline scenario, shifting the wood fiber use from pulp and paper to longer-lived products would extend the retention period of carbon in HWPs given that pulp and paper products have a much shorter lifetime than solid wood products. More benefits may be gained from displacement effects due to substitution of fossil-intensive products. Since this strategy only changes the wood commodity mix and does not affect the harvest level, it can be combined with all forest management strategies discussed previously to seek more mitigation benefits.
